# Bacterial profile, antimicrobial susceptibility patterns, and associated factors among bloodstream infection suspected patients attending Arba Minch General Hospital, Ethiopia

**DOI:** 10.1038/s41598-021-95314-x

**Published:** 2021-08-05

**Authors:** Melkam Birru, Melat Woldemariam, Aseer Manilal, Addis Aklilu, Tsegaye Tsalla, Asaye Mitiku, Tigist Gezmu

**Affiliations:** Department of Medical Laboratory Science, College of Medicine and Health Sciences, Arba Minch University, Arba Minch, Ethiopia

**Keywords:** Microbiology, Diseases, Health care, Medical research

## Abstract

Bacterial bloodstream infections are of great concern globally. Of late, the emergence of drug resistant bacteria worsen the related morbidity and mortality. This study was aimed to determine the bacterial profile, antimicrobial susceptibility patterns, and associated factors among the blood stream infection (BSI) suspected patients attending the Arba Minch General Hospital (AMGH), southern Ethiopia, from 01 June through 31st August, 2020. A cross-sectional study was conducted among 225 BSI suspected patients. Systematic random sampling method was used to select patients. Blood culture was done to isolate bacterial pathogens. Antimicrobial susceptibility test was performed by employing the Kirby-Bauer disc diffusion method. Descriptive statistics and multivariable logistic regression analysis were done by Statistical Package for Social Service (SPSS) version 22. The rate of prevalence of bacteriologically confirmed cases was 22/225 (9.8%). Majority of BSI were caused by Gram-positive cocci, 13/22 (59.1%), particularly the isolates of *S. aureus,* 7/22 (31.8%) followed by *Enterococci* species, 4/22 (18.2%) and coagulase-negative *Staphylococci* (CoNS), 2/22 (9.1%). Among the Gram-negative bacteria 9/22 (41.1%), *Klebsiella* species 4/22 (18.2%) was the prominent one followed by *Escherichia coli* 2/22 (9.1%), *Pseudomonas aeruginosa* 2/22 (9.1%), and *Enterobacter* species 1/22 (4.5%). All the isolates of Gram-negative bacteria were susceptible to meropenem whereas 69.2% of the isolates of Gram-positive counterparts were susceptible to erythromycin. Slightly above two third (68.2%) of the total isolates were multidrug resistant. Insertion of a peripheral intravenous line was significantly associated with BSI [*p* = 0.03; Adjusted Odds Ratio = 4.82; (Confidence Interval: 1.08–21.46)]. Overall results revealed that eventhough the prevalence of BSI in Arba Minch is comparatively lower (9.8%), multidrug resistance is alarmingly on the rise, which is to be addressed through effective surveillance and control strategies.

## Introduction

Blood stream infections (BSI) can be caused by bacteria, fungi, viruses, and protozoa. Among the four groups of pathogens, bacteria accounts for the majority of BSI^1^. Bactermia is defined as the presence of viable bacteria in blood without any multiplication and it may appear as transient, continuous or intermittent. Septicemia is a condition in which bacteria multiply in an active manner and circulate in the blood stream and may produce toxins that inflict harm to various organs of the host. According to the severity and the extent of organ failure; BSI can be classified into two stages such as sepsis and septic shock. *Staphylococcus aureus*, CoNS, *Enterococci,* and alpha-hemolytic *Streptococci* are the most important and most common species of Gram-positive bacteria that can enter the bloodstream. Other important types include Gram-negative bacteria such as *Escherichia coli, Klebsiella pneumoniae, Pseudomonas aeruginosa, Salmonella typhi,* and *Acinetobacter* species The geographical distribution of these bacteria vary from place to place^2^.

Blood stream infections caused by bacteria range from self-limiting to life-threatening^3^. Microbial invasion of the bloodstream can have very serious immediate consequences such as shock, multiple organ failures, and disseminated intravascular coagulopathies^4^. Changing patterns of epidemiology, lack of proper antimicrobial guidelines in the locality, the emergence of antimicrobial resistance, and paucity of good diagnostic facilities are the major factors connected to the surge in BSI associated morbidity and mortality^5^.

Studies had revealed that the number of cases of BSI are increasing worldwide^6,7^. Blood stream infection caused by bacteria are among the main causes of mortality and morbidity across the globe^8^, particularly, the mortality range from 4 to 41.5% depending on the severity, age, sex, and other risk factors involved^9^. Besides, high incidence rates of BSI were seen during advanced age due to weak immuno-competency, most often clubbed with some co-morbid conditions^10^. In Europe and North America, population-based studies had estimated 1,200,000 and 575,000–677,000 episodes of BSI per year^6^. Besides, a recent meta-analysis study revealed that the prevalence of community-onset BSI was 14.6% (range, 3.4 to 38.2%) in Africa, 7.3% (range, 2.0 to 48.4%) in Asia, 2.9% (range, 2.1 to 19.2%) in Europe, and 7.3% (range, 2.9 to 15.6%) in the Americas^11^. Blood stream infections are also the leading causes of morbidity and mortality among adults in sub-Saharan Africa^12^. It has been reported that in sub-Saharan countries, fatality owing to BSI in hospitals is very high, ie., 39%^13^. The rates of prevalence of BSI are documented to range from 11 to 28% in Eastern African countries^13–15^. In Ethiopia, the pooled prevalence of BSI was relatively lower, ie., 27.78%, among a diverse study population^16^. An earlier study conducted in the national level in the country also indicated that the overall mortality rate due to BSI was only 15.1%^17^. Also in Addis Ababa, the capital city, patients with positive blood culture had a fivefold more death rate than their culture-negative counterparts^18^.

Treatments of infections caused by BSI have become extremely challenging for physicians, particularly in African countries where a definitive therapy is only occasional^19^ and Ethiopia is of no exception. Over the counter supply and arbitrary usage of antibiotics without proper microbiological tests for drug resistance, lack of knowledge about the extent of drug resistance prevailing in the society, and unregulated sales of antimicrobials, most often for self-treatment, (without prescriptions), are leading to the rapid spread of resistant bacteria in the country^19^. The threat from MDR bacteria is presently growing in Ethiopia and it needs immediate attention^20^. An excellent review which appeared five years ago comprehensively described the epidemiology of BSI and the antibiotic resistance in on a global level, including Africa^21^. However, it is not possible to arrive at a definite conclusion regarding the patterns or the extent of bacterial resistance causing BSI, in an Ethiopian context from the published literature. Bacterial BSI exist among diverse populations from various geographical locations of the country^17,18^.

Cases of BSI are slowly on the rise in AMGH (personnel communication with clinicians) and due to the lack of prompt culture facilities, treatments are most often decided exclusively based on clinical criteria. Empirical treatment inclusive of the first line drug (ceftriazone), and if needed, the second line drugs meropenem and vancomycin are the only options available for the management of BSI in the study area. Factors associated with BSI in Ethiopia, inclusive of Arba Minch, still remain overlooked. Therefore, this study is aimed to determine the bacterial profile, antimicrobial susceptibility patterns, and associated factors among BSI suspected patients attending the Arba Minch General Hospital (AMGH), southern Ethiopia.

## Materials and methods

### Study area and period

The study was conducted at AMGH in Gamo Gofa Zone from 01st June through 31st August 2020. As per the annual report (2019) of Zonal Health department, the total population is 1, 544, 752. The hospital is situated in Arba Minch town of Southern Nations, Nationalities and Peoples’ Region and located 505 km south of Addis Ababa. Arba Minch General Hospital is currently serving more than 1.5 million people per year and providing preventive, rehabilitative, and curative care through several departments. The in-patient wards are serving more than eleven thousand while the emergency outpatient department (OPD) is treating about seven thousand patients annually.

### Study design and population

A cross-sectional study was conducted at five different wards of AMGH, comprising all patients clinically suspected of BSI (those who have body temperature greater than 38 °C, having tachycardia/tachypnea, leukocytosis/leukocytopenia) who fulfill the inclusion criteria. The inclusion criteria for the study subjects were (1) all patients ≥ 18 years who attended the OPD during the study period with suspected BSI, (2) those who were already admitted and who are suspected to have BSI. The exclusion criteria were: (1) those who took antibiotics 72 h prior to sample collection and (2) those who were in a state of coma. The study has been ethically approved by the Institutional Review Board of the College of Medicine and Health Sciences, Arba Minch University (Ref. IRB/176/12/17/03/2020).

### Sample size determination and sampling technique

The required sample size was calculated using a single population proportion formula. A prevalence of 15.8% was chosen from a previous study conducted in adults having BSI, Jimma, Ethiopia^22^. After considering a confidence interval of 95% (z = 1.96) and 5% of marginal error (d = 0.05), the initial sample size was estimated to be 204 and by computing a 10% non-response rate, the final sample size was consolidated as 225 (n). The average values of ‘n’ obtained in the previous year (2019), during three consecutive months (June 1st through August 31st) were 29 (OPD), 143 (internal medicine), 60 (surgery), 20 (ICU), and 48 (obstetrics and gynaecology). The sample size for each ward was calculated according to the proportion formula and is equal to:

$$ \frac{{{\text{Number of BSI suspected patients from a particular ward}} \times {\text{ sample size}}}}{{\text{Total population}}} $$

The final sample size proportionately attained for each ward was 22 (OPD); 36 (obstetrics and gynaecology); 15 (ICU); 107 (internal medicine) and 45 (surgery). A systematic random sampling technique was used to select the study participants by calculating the *K* th value and it was inferred from the number of BSI suspected patients who attended the respective wards. The *K* th value was calculated as 300/225 = 1.3 and hence the patients were selected in a consecutive fashion.

### Operational definition of BSI suspected patients

A patient whose age was > 18 and was showing atleast two or more clinical signs and symptoms, which include fever or hyperthermia, leukopenia or leukocytosis, tachycardia, and tachypnea with suspected or defined sites of infection, was considered a BSI suspect.

### Data collection

Written informed consents were obtained from each participant. All of them were examined by trained physicians. A pre-tested semi-structured questionnaire was used to collect the socio-demographic and socio-economic factors (age, sex, marital status, residence, educational status, occupation, family size and monthly income) through a face-to-face interview, and the clinical data of each patient were obtained from medical records.

### Blood sample collection, culture and identification

Ten ml of blood was withdrawn twice aseptically by using a butterfly vacutainer from two separate peripheral veins maintaining a time gap of 30–60 min which was then directly inoculated into the blood culture bottle containing 100 ml Tryptic Soy Broth (TSB) in 1:10 ratio, from patients in the respective wards of AMGH. Samples were then transported to the nearby Medical Microbiology and Parasitology Laboratory, Department of Medical Laboratory Science, College of Medicine and Health Sciences, Arba Minch University and immediatelyincubated aerobically at 37 °C. Routine inspections were done twice a day, for a week for the presence of bacterial growth, like uniform/subsurface turbidity, hemolysis, coagulation of broth, surface floccular deposit, pellicle formation, and gas production, . They were further examined by Gram staining and then sub-cultured aseptically on blood agar, chocolate agar, MacConkey agar, and mannitol salt agar. The chocolate agar plates were incubated inside a candle jar to provide 5–10% CO_2_, whereas the other three agar plates (blood agar, MacConkey agar, and mannitol salt agar) were incubated aerobically for 18–24 h at 37 °C.

Growths if found in both the bottles were interpreted as positive whereas growth in any one of the blood culture bottles was declared as contamination (pseudobacteremia)^22^. Blood culture bottles with no signs of bacterial growth were similarly sub-cultured in the above mentioned pairs of agar media, after a week and were considered culture-negative if there were no growths^23^. Pure cultures of bacterial isolates were subsequently subjected to species identification and confirmation. Morphological, biochemical, and physiological characteristics of isolated bacteria were ascertained by adopting laboratory methods^24^. Briefly mentioning, Gram-positive isolates were identified using catalase and coagulase tests. Isolates of members of Enterobacteriaceae family were identified biochemically by means of a series of tests: catalase, indole, citrate, urease, H_2_S production, methyl red, Voges–Proskauer, and triple-sugar iron. Non–lactose fermenting Gram-negative bacteria were identified by indole, triple-sugar iron, urease oxidase, and catalase tests^25^.

### Antimicrobial susceptibility testing

Antibiotic susceptibility profile was determined by employing the Kirby-Bauer disc diffusion technique according to the criteria set by the Clinical Laboratory Standard Institute (CLSI)^26^. For the assay, inoculums of respective bacteria were prepared in a sterile normal saline in such a way to maintain equivalent density as per the 0.5 McFarland standard. Test organisms were uniformly swabbed over the Mueller–Hinton agar and exposed to a concentration gradient of antibiotic diffusion, and then incubated at37°C for 24 h.

Fifteen commercially available antibiotic discs (Oxoid, Basingstoke, Hampshire, UK) were used. For Gram-positive bacteria, penicillin (P) (10 μg), cefoxitin (FOX) (30 μg), chloramphenicol (CHL) (30 μg), tetracycline (TC) (30 μg), doxycycline (DOX) (30 μg), vancomycin (VAN) (30 μg), erythromycin (ERY) (15 μg), gentamicin (CN) (10 μg), and ciprofloxacin (CIP) (5 μg) were used. In the case of Gram-negative bacteria, ampicillin (AMP) (10 μg), piperacillin (PIP) (100 μg), ceftriaxone (CRO) (30 μg), cefepime (CFP) (30 μg), amoxicillin-clavulanate (AUG) (20 μg), gentamicin (CN) (10 μg), tetracycline (TC) (30 μg), chloramphenicol (CHL) (30 μg), ciprofloxacin (CIP) (5 μg), and meropenem (MEM) (10 μg) were chosen. Antibiotics were selected based on the prescription policy followed in AMGH, which is the same as the national policy and also as per the recommendations of CLSI, 2019^26^. Diameters of the zones of inhibition were measured to the nearest millimeter and categorized as sensitive, intermediate, and resistant according to CLSI^26^. Isolates were classified as either susceptible or resistant to an antibiotic and all isolates with intermediate resistance were classified as resistant for a better fit in further statistical analysis.

### Detection of multiple drug resistance and Methicillin-resistant *S. aureus*

The multi-drug resistance (MDR) in this study was extrapolated as resistance to three or more groups of antibiotics tested^27^. Isolates of *S. aureus* were further tested for methicillin resistance according to the CLSI guidelines by using cefoxitin disc^26^.

### Quality control

Prior to data collection, training was given to data collectors. A pretest was conducted in 5% (n = 11) of study participants at Chencha hospital to guarantee the quality. One day training was given for data collectors to minimize inter personal variations in the identification of clinical cases. The data were checked for completeness, accuracy, clarity, and consistency by the investigator on a daily basis. To maintain the quality, standard operating procedures (in-house SOP manual) were followed during collection (aseptic technique), transportation, and processing. Before sample processing, the prepared culture media were checked for sterility by incubating five percent of it for overnight and examining the presence of any growth. Fitness of the media were checked by inoculating control strains, before culture and sensitivity tests were also performed. Control slides were used to check the quality of Gram staining. Corresponding American Type Culture Collection (ATCC) strains were used as reference (standard) for quality control related to culture and susceptibility tests; *S. aureus* (ATCC 25923), *E. coli* (ATCC 25922), and *P. aeruginosa* (ATCC 27853). All the reference strains were procured from Ethiopian Public Health Institute.

### Data analysis

The data were analyzed using SPSS, Chicago, IL, the USA for Windows, version 22. Descriptive statistics, including frequency, mean and standard deviationswere done. Bivariate and multivariate logistic regression analyses were performed to evalaute the association between variables and the BSI. Variables with a *p*-value < 0.25 in the bivariable logistic regression model were subsequently analyzed in the multivariable logistic regression to control the confounding factors, and a *p*-value ≤ 0.05 from multivariable logistic regression was considered statistically significant^28^.

## Results

### Socio-demographic and socio-economic characteristics

A total of 225 (*n*) patients suspected of BSI were included in this study with zero non-response rate. The proportion of male patients was 115/225 (51.1%) with an overall sex distribution ratio of almost 1:1. The mean age (± SD) of participants was 47 ± 13.8, whereas the actual age ranged between 19 and 78; 98/225 (43.6%) of the participants were in the age group of 41–59. Urban residents and government employees accounted for 140/225 (62.2%) and 85/225 (37.8%), respectively. Most of the participants 157/225 (69.8%) were married. Participants with higher educational qualifications (university/college/diploma) correspond to 94/225 (41.8%). Tachypnea was observed in majority 199/225 (88.4%) of cases, followed by leukocytosis 180/225 (80%) and tachycardia 158/225 (70.2%). Abnormal temperature were observed in -173/225 (76.9%) of patients (Table 1).

**Table 1:** Socio-demographic, socio-economic and clinical characteristics among BSI suspected patients (n = 225) at AMGH, southern Ethiopia, 2020.

Variables	Category	Frequency	Percentage (%)
**Socio-demographic and socio-economic characteristics**			
Sex	Female	111	49.3
	Male	114	50.7
Age	18–40	63	28
	41–59	98	43.6
	≥ 60	64	28.4
Marital status	Married	157	69.8
	Single	50	22.2
	Divorced	12	5.3
	Widowed	6	2.7
Residence address	Urban	140	62.2
	Rural	85	37.8
Educational status	Write and read only	87	38.7
	Primary (1–8)	13	5.8
	Secondary (9–12)	31	13.8
	University/college/diploma	94	41.7
Occupation	House wife	66	29.3
	Farmer	45	20
	Government employee	85	37.8
	Student	15	6.7
	Merchant	14	6.2
Family size	< 3	81	36
	3–5	89	39.6
	> 5	55	24.4
Monthly income (Ethiopian Birr)	< 1000	65	28.9
	1000–2000	81	36
	> 2000	79	35.1
Clinical Criteria	**Body temperature (°C)**		
	> 38	91	40.5
	< 36	82	36.4
	**Heart rate in beat/min**		
	Tachycardia	158	70.2
	Respiratory rate in breath/min	199	88.4
	Tachypnea		
	WBC in cells/ml		
	Leukocytosis	180	80
	Leukocytopenia	7	3.1

BSI: Bloodstream infections, AMGH: Arba Minch General Hospital, WBC: White blood cell, ml: milliliter.

### Prevalence of bloodstream infections

Out of the 225 blood samples processed for culture, 22 (9.8%) culture sets which showed bacterial growth had bacteriologically confirmed BSI. No growth was observed in 198/225 (88%) blood culture sets. Five (2.2%) of the positive results were contaminanted and therefore exempted from further analysis. Amongst the culture-positives (n = 22), 13 (59.1%) were males. The age groups 18–40 and 41–59 had the highest number of positive cases, ie., 10 (45.5% in each group). In the present study, maximum number of culture-positive cases were observed among patients admitted in the departments of internal medicine 9/22 (40.9%), followed by surgical 5/22 (22.7%), ICU 5/22 (22.7%), OPD 2/22 (9.1%), and obstetrics and gynaecology 1/22 (4.5%). Irrespective of the wards, the highest number of bacterial isolates were found among patients having infections in their respiratory tract, ie., 7/22 (31.8%), followed by genito-urinary tract 6/22 (27.3%). However, the occurrence of intra-abdominal foci was not that frequent, ie., 3/22 (only 13.7%). The most frequent co-morbid conditions observed among culture-positive patients were diabetes mellitus, ie., 5/22 (22.7%), followed by human immunodeficiency virus infection (HIV) 4/22 (18.1%), chronic obstructive pulmonary diseases 2/22 (9.1%), renal failure 2/22 (9.1%), liver diseases, malnutrition, heart failure, and multiple conditions 1/22 (4.5% for each). With respect to various medical procedures associated with culture-confirmed patients, the process of urinary catheterization was the most frequently done, 6/22 (27.3%), followed by the insertion of peripheral intravenous line, 3/22 (13.6%) and surgery 3/22 (13.6%).

### Bacteriological profiles of culture-positive patients

**Figure 1:**
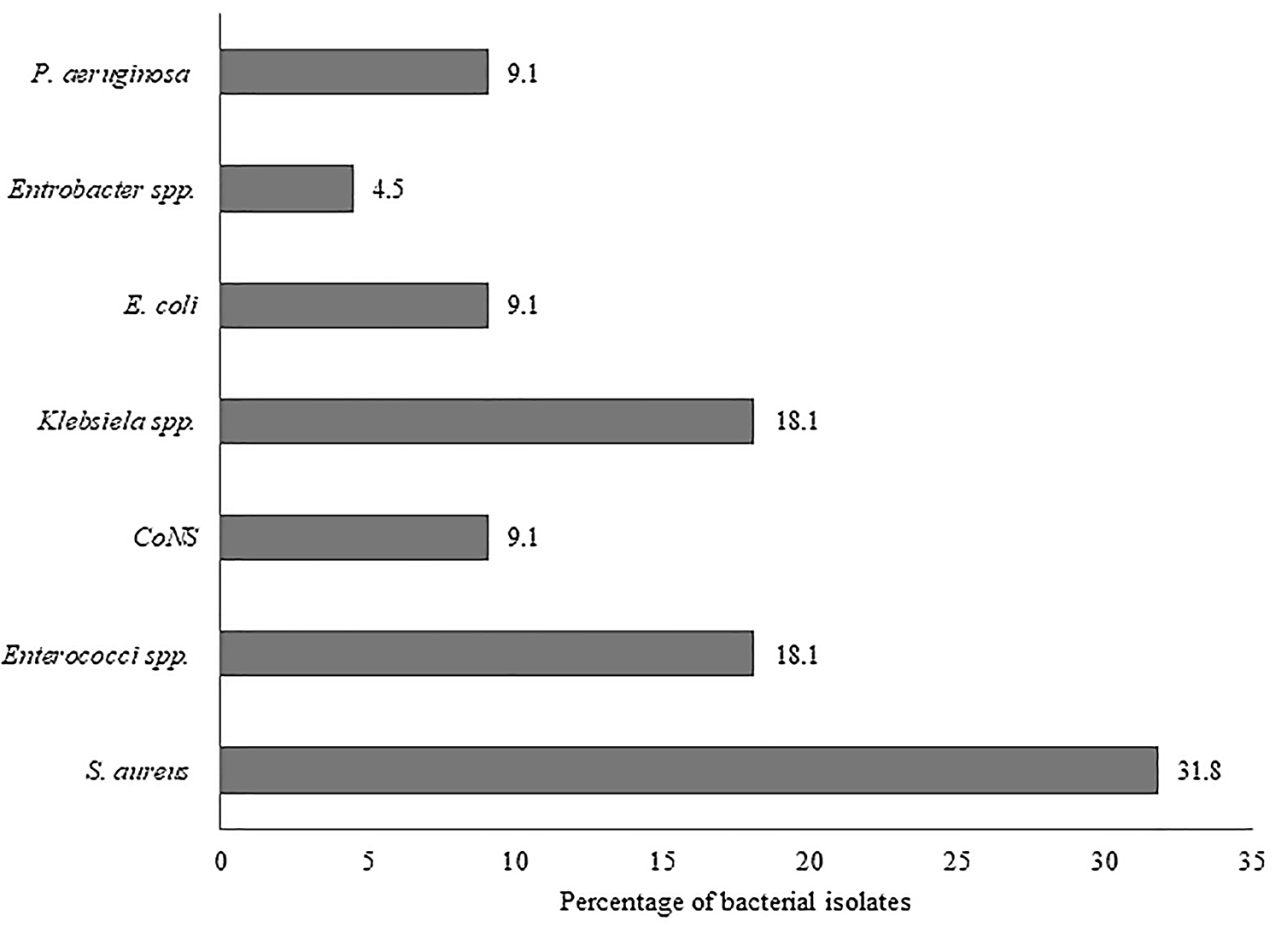
Types and percentage of blood culture isolates among adult patients at AMGH, south Ethiopia, from 1st June to 31st August, 2020.

A total of 22 (*n*) bacterial pathogens were isolated from 225 blood culture sets (Table 2). Majority 13/22 (59.1%) of the isolates responsible for BSI was Gram-positives (*S. aureus*, CoNS and *Enterococci* species) whereas Gram-negatives (*Klebsiella* species, *P. aeruginosa, E. coli* and *Enterobacter* species) accounted 9/22 (40.9%). In the case of Gram-positive bacteria, *S. aureus* 7/22 (31.8%) showed a slight predominance followed by *Enterococci* species, 4/22 (18.1%) and CoNS 2/22 (9.1%), while *Klebsiella* species was the most frequently obtained, 4/22 (18.1%), followed by *E. coli* 2/22 (9.1%) and *Enterobacter* species 1/22 (4.5%). It is to be noted that only monobacterial infection was observed in all cases (Fig. 1).

**Table 2:** Bacterial isolates of blood culture among patients attending at AMGH, southern Ethiopia, 2021.

Gram-reaction	Isolated species	Frequency	Percentage (%)
Gram-positive bacteria	*S. aureus*	7	31.8
	CoNS	2	9.1
	*Enterococcus* species	4	18.2
Gram-negative bacteria	*Klebsiella* species	4	18.2
	*E. coli*	2	9.1
	*P. aeruginosa*	2	9.1
	*Enterobacter* species	1	4.5

### Distribution of isolated bacteria

In connection with the site of infections, Gram-positive cocci, *S. aureus* was the predominant one (n = 7), with a distribution, respiratory tract (n = 2), genitourinary tarct (n = 2), surgical site (n = 1) and multiple source (n = 1). However, in the case of Gram-negative isolates, *Klebsiella* species (n = 4) was the most common, which distributed in respiratory tract (n = 3) and genitourinary tract (n = 1).

With regard to the co-morbid conditions, Gram-positive isolate, *S. aureus* (n = 5) showed a preponderance, ie., n = 5. It was distributed in such a proportion that in patients with diabetic mellitus, n = 2, COPD, n = 2 and liver diseases, n = 1. However, in the case of Gram-negative isolates, *Klebsiella* species exhibited a predominance (n = 4), with a distribution pattern of two in HIV patients and one each in those suffering from diabetis mellitus and renal disease.

In culture-positive patients, in asscoiation with the various medical procedures, *S. aureus* (n = 4) was the dominant isolates, just like in the above discussed cases of clinical charateristics. Two isolates of it was found in te patients with peripheral intravenous lines and urinary catheterization. Mentioning the case of Gram-negatives, *Klebsiella* species was the pronounced isolate (n = 3), with two in patients with urinary catheterization and one each in the case of a patient under mechanical ventilation.

### Antimicrobial resistance/susceptibility patterns of blood culture isolates

#### Gram-positive isolates

Susceptibility patterns of Gram-positive bacteria (n = 13) isolated from the blood cultures of suspected with BSI against nine antibiotics were presented in Table 3. Gram-positive isolates produced wider variations in their resistance/susceptibility patterns. The extent of resistance of Gram-positive isolates ranged between 25 and 76.9%. Most of these isolates, ie., 10/13 (76.9%) and 8/13 (61.5%) were resistant against penicillin and doxycycline respectively. Lower or medium percentage of resistance only were produced by the bacteria ie., 1/4 (25%), 4/13 (30.7%), 3/9 (33.3%), 4/9 (44.4%), 4/9 (44.4%) and 6/13 (46.1%) against a series of antibiotics such as vancomycin erythromycin gentamicin tetracycline ciprofloxacin and chloramphenicol respectively. The intermediate patterns were considered as resistant.

**Table 3:** Antimicrobial susceptibility patterns of bacterial isolates from blood cultures of patients attending at AMGH, southern Ethiopia.

Bacterial species	Total n (%)	S R n (%)	Antimicrobial agents tested														
			**P**	**FOX**	**TC**	**CN**	**ERY**	**CIP**	**DOX**	**CHL**	**VAN**	**AMP**	**PIP**	**CRO**	**CFP**	**AUG**	**MEP**
*S. aureus*	7 (31.8)	S R	2 (29) 5 (71)	3 (43) 4 (57)	4 (57) 3 (43)	4 (57) 3 (43)	5 (71) 2 (29)	4 (57) 3 (43)	3 (43) 4 (57)	4 (57) 3 (43)	NT	NT	NT	NT	NT	NT	NT
*Enterococci* spp.	4 (18.2)	S R	1 (25) 3 (75)	NT	NT	NT	2 (50) 2 (50)	NT	1 (25) 3 (75)	2 (50) 2 (50)	3 (75) 1 (25)	NT	NT	NT	NT	NT	NT
CoNS	2 (9.1)	S R	‒ 2 (100)	1 (50) 1 (50)	1 (50) 1 (50)	2 (100) ‒	2 (100) ‒	1 (50) 1 (50)	1 (50) 1 (50)	1 (50) 1 (50)	NT	NT	NT	NT	NT	NT	NT
*Klebsiella* spp.	4 (18.2)	S R	NT	NT	2 (50) 2 (50)	3 (75) 1 (25)	NT	3 (75) 1 (25)	NT	1 (25) 3 (75)	NT	1 (25) 3 (75)	NT	1 (25) 3 (75)	2 (50) 2 (50)	1 (25) 3 (75)	4 (100) ‒
*E. coli*	2 (9.1)	S R	NT	NT	1 (50) 1 (50)	1 (50) 1 (50)	NT	2 (100) ‒	NT	1 (50) 1 (50)	NT	‒ 2 (100)	NT	‒ 2 (100)	2 (100) ‒	1 (50) 1 (50)	2 (100) ‒
*P. aeruginosa*	2 (9.1)	S R	NT	NT	NT	1 (50) 1 (50)	NT	1 (50) 1 (50)	NT	NT	NT	NT	‒ 2 (100)	NT	1 (50) 1 (50)	NT	2 (100) ‒
*Enterobacter* spp.	1 (4.5)	S R	NT	NT	1 (100) ‒	1 (100) ‒	NT	1 (100)	NT	1 (100) ‒	NT	‒ 1 (100)	NT	1 (100) ‒	1 (100) ‒	1 (100) ‒	1 (100) ‒
Total isolates	22 (100)	S R	3 (23.1) 10 (76.9)	4 (44.5) 5 (55.5)	9 (56.3) 7 (43.7)	12 (66.6) 6 (33.83)	9 (69.2) 4 (30.7)	12 (66.7) 6 (33.3)	5 (38.5) 8 (61.4)	10 (50) 10 (50)	3 (75) 1 (25)	1 (14.3) 6 (85.7)	‒ 2 (100)	2 (28.6) 5 (71.4)	6 (66) (33)	3 (42.9) 4 (57.1)	9 (100) ‒

S: susceptible, R: resistant, NT: not tested.

P: penicillin, FOX: cefoxitin, CHL: chloramphenicol, ERY: erythromycin, CN: gentamicin, TC: tetracycline, CIP: ciprofloxacin, VAN: vancomycin, DOX: doxycycline, AMP: ampicillin, PIP: piperacillin, CRO: ceftriaxone, AUG: amoxicillin-clavulanate, MEM: meropenem, CFP: cefapime. *NT corresponds to a change in denominator (total number of isolates tested).

Isolates of *S. aureus* were resistant to antibiotics such as penicillin 5/7 (71.1%), and doxycycline 4/7 (57.1%). Among the seven isolates of *S. aureus*, four were with a zone of inhibition ≤ 21 mm (16.2 to 19.5 mm)), in the cefoxitin disc diffusion assay and were extrapolated as methicillin-resistant *S. aureus* (MRSA). However, a lower resistance was produced against tetracycline 3/7 (43%), gentamicin3/7 (43%), chloramphenicol 3/7(43%), ciprofloxacin 3/7 (43%), and erythromycin 2/7(29%). The percentage of MRSA among *S. aureus* was 4/7 (57.1%).

Only one of the isolates of CoNS, 1/2 (50%) was methicillin-resistant whereas all others showed susceptibility towards both gentamicin and erythromycin. However, all these isolates exhibited resistance to penicillin, and 1/2 (50%) of them were resistant against tetracycline, chloramphenicol, doxycycline, and ciprofloxacin.

In the case of *Enterococci* species, 3/4(75%) of the isolates were resistant against penicillin and doxycycline. Furthermore, 2/4(50%) of these isolates only exhibited resistance against chloramphenicol and erythromycin whereas 1/4(25%) showed resistance to vancomycin.Overall susceptibility patterns of Gram-positive isolates revealed that 9/13(69.2) and 6/9(66.7%) respectively were susceptible to erythromycin and gentamicin (excluding *Enterococci*). On the other hand, 10/13(76.9%) and 8/13(61.5%) of isolates were resistant against penicillin and doxycycline respectively.

#### Gram-negative isolates

The extent of antimicrobial resistance by Gram-negative organisms are shown in Table 3. These organisms exhibited considerable resistance ie., 6/7 (85.7%), 5/7 (71.5%), 4/7 (57.1%) and 4/7(57.1%) against ampicillin ceftriaxone chloramphenicol and amoxicillin-clavulanate respectively At the same time, a lower percentage of resistance, ie., 2/9 (22.2%), 3/9 (33.3%), 3/7 (42.8%), and 3/9 (33.3%) were produced against ciprofloxacin, gentamicin, tetracycline and cefepime respectively. All of these Gram-negative isolates were susceptible to meropenem.

All the isolates of *P. aeruginosa* were found to be resistant to piperacillin, 2/2 (100%), and on the contrary, all were susceptible to meropenem, 2/2 (100%). Similarly, isolate of *Enterobacter* species had shown resistance to ampicillin 1/1 (100%), but it issusceptible to ciprofloxacin, chloramphenicol, tetracycline, gentamicin, ceftriaxone, cefepime, amoxicillin-clavulanate and meropenem. Hundred percentage of *E. coli* were resistant to both ampicillin and ceftriaxone*.* Furhtermore*,* it was also observed that all these isolates were susceptible to cefepime, ciprofloxacin, and meropenem. Three fourth (75%) of the species of *Klebsiella* isolates were resistant to ampicillin, ceftriaxone, chloramphenicol, and amoxicillin-clavulanate. However, 75% of the isolates were susceptible to gentamicin and ciprofloxacin while all of them showed susceptibility to meropenem (Table 5).

Altogether, it is clear from the results that 9/9(100%) and 7/9 (77.7%) isolates of Gram-negative bacilli respectively were susceptible to meropenem and ciprofloxacin. At the same time 6/7(85.7%) and 5/7(71.4%) of isolates respectively were resistant against ampicillin and ceftriazone (excluding isolates of *Pseudomonas* species).

### Multi-drug resistance

The most common antimicrobial resistance patterns observed are presented in Table 4. Out of the 22 (n) total bacterial isolates, MDR was found in 15 (68.2%). Nine isolates (69.2%) belong to the Gram-positive catogery and five (66.7%) were Gram-negatives. The MDR patterns of Gram-positive bacteria consist of 4/7 (57.1%) of *S. aureus*, 3/4 (75%) of *Enterococci* species *.* and 2/2 (100%) of coagulase-negative *Staphylococci.* MDR patterns of Gram- negative bacteria comprise of 3/4 (75%) of *Klebsiella* species, 1/2 (50%) of *P. aeruginosa* and 2/2 (100%) of *E. coli.*

**Table 4:** Multiple antibiotic resistance patterns of bacterial isolates from blood culture of patients at AMGH, southern Ethiopia 2020.

Bacterial isolates	Resistance patterns						
	R0	R1	R2	R3	R4	> R5	MDR (%)
*S. aureus* 7 (31.8)	–	–	3 (42.8%)	–	1 (14.2%)	3 (42.8%)	4 (57%)
*Enterococcus* 4 (18.2)	1 (25%)	–	–	2 (50%)	–	1 (25%)	3 (75%)
CoNS 2 (9.1)	–	–	–	1 (50%)	–	1 (50%)	2 (100%)
Gram-positive bacteria 13 (59.1)							9 (69.2%)
*Klebsiella*spp*.* 4 (18.2)	–	1 (25%)	–	–	1 (25%)	2 (50%)	3 (75%)
*E. coli* 2 (9.1)	–	–	–	1 (50%)	–	1(50%)	2 (100%)
*P.aeruginosa* 2 (9.1)	–	–	1 (50%)	1(50%)	–	–	1 (50%)
*Entrobacter*spp*.* 1 (4.5)	–	1 (100%)	–	–	–	–	–
Gram-negative bacteria 9 (40.9)							5 (66.7%)

n: number of isolates, R0: No resistance at all, R1: resistant to one antibiotic, R2: resistant to two antibiotics, R3: resistant to three antibiotics, R4: resistant to four antibiotics, R5: resistant to five or more antibiotics.

### Factors associated with bloodstream infections

In bivariable logistic regression analysis, BSI was statistically significant in patients with age ≥ 60 (*p* = 0.03), and in those who underwent a medical procedure (incorporation of a peripheral intravenous device (*p* = 0.02) and or any surgery (*p* = 0.04)). However, in multivariable logistic regression analysis, only having a peripheral intravenous device (*p* < 0.05) had shown a statistical significance with BSI (Table 5). The odds of BSI are 4.82 times higher in patients having a peripheral intravenous device compared with those who do not have it (*p* = 0.03; AOR = 4.82; CI = 1.08–21.46).

**Table 5:** Bivariable and multivariable logistic regression analyses of factors associated with BSI among suspected patients at AMGH, southern Ethiopia, 2020.

Characteristics	Blood culture result		Bivariable analysis		Multivariable analysis	
	Positiven(%)	Negativen(%)	*p*-value	COR (95% CI)	*p*-value	AOR (95% CI)
**Sex**						
Male	13 (11.3)	102 (88.6)	0.41	1.43 (0.59–3.56)		
Female	9 (8.1)	101 (91.8)	1	1		
**Age**						
18–40	10 (14.9)	57 (85.1)	1	1		
41–59	10 (10.4 )	86 (89.6)	0.027*	0.66 (0.036–0.815)		3.528
≥ 60	2 (3.2)	60 (96.5)	0.112	0.19 (0.060–1.341)	0.2	(0.514–24.228)
**Marital status**						
Married	13 (8.2)	144 (91.7)	1	1		
Single	4 (8)	46 (92)	0.483	0.96 (0.24–20.4)		
Divorced	4 (33.3)	8 (66.6)	0.492	5.53 (0.21–24.7)		
Widowed	1 (16.7)	5 (83.3)	0.465	2.21 (0.034–4.6)		
**Residency**						
Rural	7 (8.2)	78 (91.7)	0.545	1.3 (0.52–3.4)		
Urban	15 (10.7)	125 (89.2)	1	1		
**Educational status**						
Write and read only	10 (11.4)	70 (80.5)	1	1		
Primary	1 (7.8)	12 (92.3)	0.75	0.58 (0.16–12.1)		
Secondary	2 (6.5)	29 (93.5)	0.44	0.48 (0.38–8.9)		
University/college	9 (9.6)	85 (90.4)	0.69	0.74 (0.46–3.1)		
**Occupational status**						
House wife	4 (6.1)	62 (93.1)	0.303	2.58 (0.4–15.7)		
Farmer	5 (11.1)	40 (88.8)	0.74	1.3 (0.2–7.7)		
Gov’t employee	9 (10.6)	76 (89.4)	0.68	1.4 (0.27–7.3)		
Student	2 (13.3)	13 (86.6)	0.941	1.08 (0.1–8.9		
Merchant	2 (14.2)	12 (85.7)	1	1		
**Site of infections**						
Respiratory tract	7 (16.6)	35 (83.3)	1	1		
Genito-urinary tract	6 (10.3)	52 (89.7)	0.378	0.57 (0.042–3.340)		
Intra-abdominal	3 (9.3)	29 (90.6)	0.718	0.51 (0.074–6.032)		
Surgical site	3 (5.7)	49 (94.3)	0.805	0.3 (0.071–7.843)		
Soft tissue and skin	1 (10)	9 (90)	0.313	0.55 (0.253–73.488)		
Multiple sources	3 (15.7)	16 (84.2)	0.678	0.93 (0.029–9.985)		
Unknown origin	1 (7.1)	13 (92.9)	0.493	0.38 (0.041–4.689)		
**Co-morbidities**						
Diabetes	5 (20)	20 (80)	1	1		
HIV	4 (21.1)	15 (78.9)	0.929	1.06 (0.046–16.744 )		
COPD	2 (16.7)	10 (83.3)	0.720	0.80 (0.048–8.201 )		
Liver disease	1 (12.5)	7 (87.5)	0.554	0.57 (0.050–4.978 )		
Renal disease	2 (13.3)	13 (86.7)	0.874	0.26 (0.063–10.478 )		
Malnutrition	1 (33.3)	2 (66.7)	0.528	2 (0.045–4.931 )		
Multiple comorbid	1 (33.3)	2 (66.7)	0.392	2 (0.010–5.985 )		
Heart failure	1 (11.1)	8 (88.9)	0.392	0.5 (0.010–5.985 )		
**Medical procedures**						
PIVL	3 (5)	57 (95)	0.028*	5.182 (1.191–22.551)	0.039**	4.822 (1.083–21.462)
Surgery	3 (5.7)	49 (94.3)	0.047*	4.455 (1.020–19.459)	0.072	
Mechanical vent	2 (28.5)	5 (71.4)	0.381	0.682 (0.105–4.432 )		3.958 (0.886–17.683)
Multiple procedures	1 (9.1)	10 (90.9)	0.688	2.727 (0.289–25.749)		
Urinary catheter	6 (21.4)	22 (78.6)	1	1		

*Statistically significant at *p* < 0.05 in bivariable analysis, **Statistically significant at *p* < 0.05, AOR: Adjusted odds ratio, COR: Crude odds ratio, 1: reference group, CI: Confidence interval. PIVL: Peripheral intravenous line, COPD: Chronic obstructive pulmonary disease, HIV: Human Immunodeficiency virus.

## Discussion

We found that the prevalence of BSI is 9.8% (95% CI; 6.1–14.1). The cuture-positivity rate is comparable to the results reported from a couple of cities in Ethiopia such as Addis Ababa (13%)^18^ and Jimma (8.8%)^29^, and also from Cambodia (8.8%)^30^. However, this prevalence was lower than that found in another study conducted in Jimma (15.8%)^22^ itself and also Mekelle (28%), Ethiopia^16^, as well as in another African country, Zambia (24%)^31^. This fluctuation in the rates of prevalence could be attributed to differences in methods employed for blood culture (manual or automated), volume of blood used (5 or 10 ml), number of blood cultures taken (2 or 3 sets), and the lack of clinical indications (variations in clinical signs and symptoms of BSI), eventually increasing the proportion of negative results^32^. Besides, the sample size, nature of patients, design of study, geographical locations, blood culturing rates as well as infection control strategies followed in different countries might have also contributed^32,33^. Above all, infections caused by anaerobes and other etiological agents would had add to this disparity, with respect to the rate of isolation of cultures.

The prevalence of BSI is found to maintain a male preponderance; however, there was no statistically significant association between the gender and BSI. A couple of studies also had documented that, majority of BSI isolates were obtained from males^34,35^. In contrast, studies reported from Mekelle and Jimma indicated that the prevalence of BSI is higher among females^16,22^.

All cases of BSI were found to be mono-bacterial and are consonant with some earlier studies reported from Addis Ababa, Ethiopia () and also from India^3,35,36^. The diversity of bacterial isolates (*S. aureus, Enterococci* species.*,* CoNS*, Klebsiella* species , *Enterobacter* species , *P. aeurogenosa,* and *E. coli*) observed in the present study is in concordance with the information from some earlier results published from USA and the Gondar, Ethiopia^37,38^.

The infectious agents responsible for BSI vary from country to country with unique geographical peculiarities^39,40^. The most commonly isolated bacterial types belong to the Gram-positive group (59.1%) and their Gram-negative counterparts correspond to 40.9%. The same trend of predominance exhibited by the Gram-positive group was also documented in previous studies reported from two cities of Ethiopia, Jimma (53%)^22^, and Mekelle (72.2%)^16^, and also from other countries, Zambia (61.7%)^31^ and USA (59%)^37^. Besides, the estimated pooled prevalence of Gram-positive and Gram-negative bacterial isolates are 15.5 and 10.4% respectively, in diverse groups of BSI suspected patients from different parts of Ethiopia^17^. However, this is in contrast to the results of other studies done in Cambodia^30^, Côte d’Ivoire^40^, and Ethiopia itself (Addis Ababa)^36^, which reported Gram-negative as the dominant group. Epidemiological variations and diversity in etiological agents might have contributed to this disparity^32,33,41^. It has been envisaged that due to the emergence of modern medical care system, Gram-positive cocci have begun to be detected often as the predominant group of pathogens causing BSI in the early 1980s itself^42^.

Among the Gram-positive group, *S. aureus* was the most prevalent (31.8%) isolate in the blood culture. Similar patterns of prevalence, in accordance with the present study were already noted in two other cities of Ethiopia viz. Addis Ababa^36^ and Jimma^22^, and also in another country, Cambodia^30^. A probable reason for the highest prevalence of *S. aureus* could be its widespread presence in hospital environments as a contaminant, which might have invaded the admitted patients, eventually establishing infections^43^. Moreover, it is also a commensal of skin and mucous membranes and may further invade the patient during surgery or instrumental manipulations^43^. However, further studies are required to arrive at a definite conclusion.

*Enterococci* species was the second most frequently isolated Gram-positive bacteria and the same trend was also observed in a previous work conducted in India too^35^. In contrast to our findings, a couple of studies reported from Ethiopia (Jimma and Addis Ababa) showed that CoNS are the second most predominant isolate among the commonly found pathogens^22,44^. These fluctuations could be correlated to the fact that during the last two decades, *Enterococci* species have been emerging as one of the major pathogens causing BSI^45^. However, in our study, CoNS was found to be a rarely isolated Gram-positive bacterium. Gram-negative bacilli, *Klebsiella* species was the most frequently isolated pathogens (18.2%) and this is in line with a previous study done in Addis Ababa, Ethiopia^44^. Bloodstream infections may arise as a primary condition or may be secondary to a focal infection at a defined body site, most commonly arising from the respiratory, gastrointestinal, and urogenital tracts^46^. In our study, a high prevalence of BSI was observed among patients having respiratory tract infections. An earlier study reported from Jimma, Ethiopia also had documented that respiratory tract is the primary site of infections^22^. These might be due to the fact that BSI are more common and possibly is the reflection of typical complications arising from community-acquired pneumonia^47^. The most prevalent co-morbid condition found in culture-positive patients was diabetes mellitus, 22.7%, and this resembles the results of some earlier studies done in Cambodia^30^ and Côte d’Ivoire^40^.

It has also been revealed that 76.9 and 61.5% of Gram-positive cocci were resistant to penicillin (and doxycycline respectively. These observations are in line with the results of several studies reported from various cities of Ethiopia (Addis Ababa, Jimma and Mekelle)^16,22,44^. In our study, most of the Gram-positive bacteria showed lower levels of percentage resistance to gentamicin (33.3%), erythromycin (30.3%), tetracycline (44.4%), ciprofloxacin (44.4%), and vancomycin (25%). The same trend of resistance was observed in several studies reported from Ethiopia (Jimma and Mekelle)^16,22^. From these results, it may be envisaged that ciprofloxacin and tetracycline are not the suitable options for an empirical therapy for BSI.

Another important aspect is that 71% of isolates of *S. aureus* had shown resistance to penicillin whereas 57% of it was resistant to doxycycline curtailing their empirical usage in the study area and this is similar to an earlier trend reported from Mekelle and Addis Ababa, Ethiopia^16,36^. Twenty nine percentage of isolates of *S. aureus* exhibited resistance to erythromycin, which is the least whereas 43% of it was resistant against each of chloramphenicol, ciprofloxacin, gentamicin, and tetracycline. This is in accordance with some earlier findings reported from Mekelle and Jimma^16,29^.

the trend observed in the current research indicated that 57.1% of *S. aureus* are MRSA strains. A similar pattern of resistance was observed in previous studies from Mekelle, Ethiopia, and USA^16,37^. The higher percentage of MRSA strains currently observed might be due to the frequent use of these drugs, especially the third-generation cephalosporin in hospital/clinics in Arba Minch as part of the emergency empirical therapy. Literature also indicated that the frequent use of cephalosporin in hospitals across the globe is correlated to the emergence and spread of MRSA^48^.

The second most predominant Gram-positive bacilli is *Enterococcus* species, 75% of which showed resistance to each of the two antibiotics, penicillin and doxycycline and these findings are similar to a previous research done in Nepal^49^. Our results also revealed that 75% of *Enterococci* species is susceptible to vancomycin. We found that all the isolates of CoNS are resistant to penicillin and this is in line with the results of a prior study conducted in Ethiopia (Addis Ababa) itself^44^. It was also observed that 50% of CoNS were methicillin resistant and this is also in agreement with a study reported from Addis Ababa^36^. On the other hand, all the isolates of CoNS were suscetible to gentamicin and erythromycin and are comparable to the results of studies done in Jimma, Ethiopia and also in India^22,50^. Besides, only 50% of the isolates showed suscetibilities towards tetracycline, doxycycline, ciprofloxacin and this is in accordance with a series of studies conducted in two cities of Ethiopia, viz. Jimma and Mekelle and also India^16,22,35^.

Gram-negative isolates showed varied patterns of resistance, ie., 85, 71 and 57% against ampicillin, ceftriaxone, chloramphenicol, and amoxicillin-clavulan respectively, ie., which are frequently used in the treatment of BSI in Arba Minch. The trend of resistance observed currently is similar to the patterns observed in a couple of previous studies conducted in Ethiopia^29,36^.

On the other hand, a lower percentage of resistance ie., 22.2, 33.3, 28.5 and 33.3% were shown against four antibiotics such as ciprofloxacin, gentamicin, tetracycline, and cefepime respectively. This resembles the results reported from Jimma and Addis Ababa^29,36,44^. It is to be specified that all the isolates of Gramnegative bacteria showed susceptibility to meropenem and a similar result was previously reported from Nepal^49^.

Three fourth (75%) of the isolates of *Klebsiella* species were resistant to ampicillin, ceftriaxone, and amoxicillin-clavulanate and on the contrary were susceptible to gentamicin and ciprofloxacin; however all the isolates were extremely susceptible to meropenem. These are comparable to the results of past studies done in Ethiopia^29,36^.

All the isolates of *E. coli* were susceptible to ciprofloxacin, cefepime, and meropenem which are by and large in line with the data obtained from a couple of studies reported from Ethiopia^29,36^. However, all of them exhibited resistance to ampicillin and ceftriaxone, and are found to be in consistency with earlier reports from Ethiopia and Nepal^22,44,49^.

Invariably all the isolates of *P. aeruginosa* were resistant to piperacillin and this is in accordance with the results of a study done in Addis Ababa^44^. On the other hand, all of them were susceptible to meropenem and is also mentioned in a work done in Nepal^49^. Isolate of *Enterobacter* species showed resistance to ampicillin and this is comparable to the results of earlier studies done in the capital city of Ethiopia^36^. Interestingly, it also showed susceptibility to all the other drugs tested.

Development of bacterial resistance against multiple drugs is a major crisis that restricts the drug of choice for the treatment of BSI. In our study, MDR was observed in the case of 68.2% of isolates, which is comparable to a study conducted in Mekelle, Ethiopia^16^. Among the MDR, 60% were Gram-positive and the rest were Gram-negative and these findings are in line with the results of a study conducted in Congo too^51^. Those authors had reported that 34.6% of the Gram-negative are MDR. Most frequently, resistance was displayed by Gram-negative bacteria against the antibiotic, ampicillin.

Among the various risk factors assessed related to BSI, insertion of a peripheral intravenous line was found to be significantly associated and it was seen that patients with this device are 4.8 times [AOR 4.82, (95 CI: 1.08–21.46)] more prone to infections. Studies so far conducted in different countries also substantiate having a peripheral intravenous line is very much a risk factor causing BSI^52,53^.

Peripheral intravenous line-associated BSI often come from either the tip of the catheter or from the skin around it, so that during insertion under emergency conditions, exogenous pathogens may enter into the bloodstream, eventually leading to BSI^54^. Another explanation is that BSI can also occur due to the lack of proper aseptic measures practiced during the process of catheterization,ie., non-compliance to catheter insertion protocols by hospital personnels.

Results of the bivariate analysis show that patients with age ≥ 60 (*p* = 0.03) and those who underwent a surgery (*p* = 0.04) were significantly associated with BSI; however, these were not identified as independent risk factors in the multivariate analysis. Likewise, no significant relationship was found among socio-demographic and socio-economic parameters, and clinical factors of patients such as the site of infections (respiratory, urinary, intra-abdominal, soft tissue and skin, and surgical), medical procedures (urinary catheterization, surgery, and mechanical ventilation), chronic diseases (diabetes mellitus, HIV, renal failure, COPD, liver disorder, and heart failure) and malnutrition.The lack of association among BSI and the aforementioned factors have also been described in studies conducted in Egypt and Japan^55,56^.

## Limitations

Shortcomings of the present work include shorter duration and the type of design of the study (cross-sectional), smaller sample size,and the lack of some of the chemicals. In addition, extended spectrum beta lactamase production among isolates and minimum inhibitory concentration for vancomycin were not detected andalso only aerobic cultures were analysed, which limit the identification of anaerobic pathogens. Also, this single-institution based study did not includea wider community and or even other hospitals. Conventional methods of blood culture were employed and finally, only the suspected cases were selected and this would probablyhad resulted in the exclusion of cases of intermittent bacteremia. Overall, this study was a single institution-based cross-sectional one and therefore the results obtained do not represent the general population. However, a remarkable finding is that the isolates of blood culture were resistant to frequently used antibiotics in Arba Minch.

## Conclusions

This study revealed that the overall prevalence rate of BSI in Arba Minch is 9.8% and it is comparatively lower than that earlier reported at the national level in Ethiopia. Gram-positive bacteria, especially the isolates of *S. aureus* were found to be the most prevalent causative agent of BSI. Based on the results of antimicrobial susceptibility tests, it might be inferred that antibiotics such as meropenem and erythromycin respectively are the effective drugs against Gram-negative and Gram-positive bacteria However, there is a notable growth in antibiotic resistance, against several clinically relevant antimicrobials (such as penicillin, ampicillin, doxycycline, amoxicillin-clavulanate, ceftriaxone, and chloramphenicol) in the study setting. A remarkably high rate (68.2%) of MDR was also observed. An important factor associated with BSI in Arba Minch is the usage of peripheral intravenous lines and it is the most mentionable finding, which is to be given due attention.
